# Lysyl Oxidase Like-4 (LOXL4) as a tumor marker and prognosticator in advanced stage laryngeal cancer^☆^

**DOI:** 10.1016/j.bjorl.2021.02.009

**Published:** 2021-03-13

**Authors:** Ozan Muzaffer Altuntaş, Nilda Süslü, Yeşim Gaye Güler Tezel, Hayriye Tatlı Doğan, Taner Yılmaz

**Affiliations:** a Koç University, Faculty of Medicine, Department of Otorhinolaryngology, Istanbul, Turkey; b Hacettepe University, Faculty of Medicine, Department of Otorhinolaryngology, Ankara, Turkey; c Hacettepe University, Faculty of Medicine, Department of Pathology, Ankara, Turkey; d Yıldırım Beyazıt University, Department of Pathology, Ankara, Turkey

**Keywords:** Larynx cancer, LOXL4 protein, Hypoxia, Organ preservation, Prognosis

## Abstract

**Introduction:**

Lysyl oxidase-like 4 is an amine oxidase from the lysyl oxidase family that was previously shown to be overexpressed in head and neck cancer and upregulated in response to hypoxia. The possible role of lysyl oxidase-like 4 as a tumor marker in advanced stage larynx cancer was investigated.

**Objective:**

To investigate the expression of lysyl Oxidase-Like 4 protein in advanced stage laryngeal cancer and elucidate its possible role as a tumor marker, predictor of treatment response and prognosticator.

**Methods:**

Diagnostic specimens of 72 patients treated for stage III–IV laryngeal squamous cell carcinoma were evaluated for lysyl oxidase-like 4 expression by immunohistochemistry.

**Results:**

Lysyl oxidase-like 4 expression was correlated with advanced tumor stage (*p* = 0.041) and better differentiation (*p* = 0.025) but was independent of tumor diameter (*p* = 0.456). Response to induction chemotherapy or the need for salvage laryngectomy were not affected by lysyl oxidase-like 4 expression (*p* = 0.999, *p* = 0.070 respectively). Increased lysyl oxidase-like 4 expression was associated with better 2 year overall survival in both univariate (*p* = 0.036) and multivariate analyses (*p* = 0.014).

**Conclusion:**

Lysyl oxidase-like 4 expression emerges with advancing stages, is lost with worsening differentiation, and may have tumor suppressive properties in larynx cancer.

## Introduction

Annually, over half a million people are diagnosed with squamous cell carcinomas (SCC) of the head and neck and laryngeal SCC (LSCC) is the second most common among these malignancies.^1^ 40% of LSCC patients present at an advanced stage and 5 year survival has exhibited a slight decline to under 60% in recent surveys.^2^ The efficacy of concomitant chemoradiotherapy for organ preservation in advanced stage LSCC was established in the RTOG 91−11 cohort with the landmark study of Forastiere, et al.^3^ However, LSCC exhibits marked clinical and genetic heterogeneity,^4^ rendering a lack of response to chemoradiotherapy, locoregional recurrence and the need for salvage surgery a significant risk. Identifying individuals that might have a poor response to organ preservation protocols or a tendency toward tumor progression despite chemoradiotherapy in LSCC via biomarkers would thus be a desirable strategy.

Tumor hypoxia is associated with aggressive biologic behavior, metastatic tendency, treatment resistance and poor prognosis in solid tumors and radiation resistance in hypoxic tumors is well established. The association between markers of hypoxia with prognosis in LSCC is a recent investigative topic that revealed a positive correlation between disease specific mortality and hypoxia markers such as carbonic anhydrase-9 (CA-9).^5^

A novel family of extracellular matrix (ECM) enzymes that catalyze the cross-linking of collagen and elastin are expressed in response to tissue hypoxia: Lysyl oxidase-like proteins (LOXLs) were proven to be essential mediators for hypoxia-induced metastasis.^6^ It was validated in the RTOG 90−03 cohort that LOX, the parent enzyme in this family, promotes metastasis and is a negative prognostic marker for survival in head and neck carcinoma.^7^ Another member of this enzyme family, LOX-like protein 4 (LOXL4, was recently established as a selective molecular marker in primary and metastatic head and neck SCC.^8^ This study aims to characterize the relationship between tissue LOXL4 expression and tumor size, nodal metastases, chemoradiotherapy response, survival in locally advanced LSCC.

## Methods

### Patient characteristics

Patients treated for American Joint Committee on Cancer (AJCC, 7th ed.) T3–4 laryngeal SCC at a tertiary care center were identified: clinicopathologic and outcomes data were recorded retrospectively. All cases were diagnosed between January 2001 – October 2013 and assigned to either total laryngectomy or organ preservation therapy with curative intent by the institutional multidisciplinary oncology board. Pretreatment formalin-fixed, paraffin-embedded tumor blocks were requested, and the diagnosis of SCC was confirmed by a senior histopathologist (G.G.T.).

The minimum follow up period was 24 months for cases without recurrence or death. Cases with insufficient follow-up data were excluded from survival analysis.

Approval for the study was obtained from the institutional ethics committee (ref. nº GO 14/4216-03) and a research grant was provided by the institutional scientific research fund.

REMARK recommendations for reporting tumor marker prognostic studies were followed.

### Immunohistochemistry

The formalin-fixed, paraffin-embedded primary laryngoscopic biopsy specimens of the patients were retrieved and 5 μm-thick sections were taken onto positively charged slides. Immunohistochemical staining was automatized using Ventana BenchMark® Gx (Roche, Basel – Sweden) automatic staining instrument. After deparaffinization at 75 °C, antigen retrieval was performed with citrate buffer for 38 min. Slides were then incubated with rabbit anti-LOXL4 antibody (Abcam, Cambridge – United Kingdom) at 5 μg/mL dilution for 20 min at 37 °C. LOXL4 positive sites were stained with 3.3’-diaminobenzidine (DAB) and counterstaining was performed with hematoxylin. Human testis and kidney tissues were used for positive controls.

### Histologic analysis

Tissue LOXL4 staining was scored by two senior histopathologists at a single session blinded to the clinical characteristics of patients. Intensity of staining was categorized as mild (1 pt.), moderate (2 pts.) and strong (3 pts.). The extent of staining was categorized as <10% (1 pt.), 11%–25% (2 pts.), 26%–50% (3 pts.), 51%–75% (4 pts.), 76%–100% (5 pts.). The multiplication product of intensity and extent of scoring was used as a composite LOXL4 expression score and cases scoring 6 points or higher (a staining of at least 11%–25% or moderate intensity) were considered LOXL4 positive for the purposes of this study. Tumor differentiation was also assessed at the same session.

### Tumor size measurement

Only for patients treated with a primary total laryngectomy, the largest diameter of the primary tumor in centimeters from the original gross pathology report was recorded.

### Statistical analysis

SPSS version 22 (IBM, Armonk – New York) was used for all statistical analyses. Categorical variables were assessed with χ^2^ testing (Fisher’s exact was substituted if expected frequencies below 20%). Comparisons between non-parametric distributions were made with Mann–Whitney *U* test and time to disease recurrence or death was estimated using the Kaplan–Meier method. Survival curves were compared with the Mantell-Cox log-rank test. Multivariate analysis for hazard estimation was done with Cox regression. All *p-*values under 0.05 were considered statistically significant. Disease free survival (DFS) was defined as time elapsed from diagnosis until any (local, regional, distant) recurrence. Overall survival (OS) was defined as time elapsed from diagnosis until death from any cause.

## Results

Seventy-two patients with T3–4 LSCC (69 males, 3 females) were included in the study. The youngest patient was 43 at the time of diagnosis and the oldest was 86 (mean age 58 [±9.4 SD]). Forty-two cases were T3 (58.3%) and 30 were T4 (41.7%) according to AJCC staging. Nodal metastases were not present in 44 patients (61.1%) yet 7 (9.7%) were N1, 19 (26.4%) were N2 and 2 (2.8%) were N3. None had distant metastases at diagnosis.

All specimens were confirmed as SCC at histopathologic analysis, with 25 (34.7%) well differentiated, 41 (56.9%) moderately differentiated, 6 (8.3%) poorly differentiated tumors.

Primary treatment for patients comprised of total laryngectomy plus neck dissection in 29 cases (40.2%). 18 cases (25%) were treated with concomitant chemoradiotherapy (70 Gy of intensity modulated radiation therapy over 35 fractions and cisplatin 35 mg/m^2^ weekly) and 21 (29.1%) required induction chemotherapy (3 cycles of cisplatin 75 mg/m^2^+ docetaxel 75 mg/m^2^ + fluorouracil 750 mg/m^2^) plus concomitant chemoradiotherapy (as described previously). The treatment of four patients (5.6%) began with induction chemotherapy but was concluded with a salvage laryngectomy due to a lack of sufficient objective response to chemotherapy in the primary tumor. Please refer to Table 1 for a summary of patient characteristics.

**Table 1:** Patient characteristics.

Characteristic		Nº of cases	Percentage of cases (%)
Gender	Male	69	95.8
	Female	3	4.2
AJCC T-classification	T3	42	58.3
	T4	30	41.7
AJCC N-classification	N0	44	61.1
	N1	7	9.7
	N2	19	26.4
	N3	2	2.8
Stage	III	32	44.4
	IVa	38	52.8
	IVb	2	2.8
Tumor differentiation	Well differentiated	25	34.7
	Moderately differentiated	41	56.9
	Poorly differentiated	6	8.3
Primary treatment	Total laryngectomy + ND	29	40.3
	Concomitant chemoradiotherapy^b^	18	25
	Induction chemotherapy^c^	25	34.7
	With response (organ preservation)	21	29.2
	No response (salvage surgery)	4	5.6

Characteristic	Mean ± SD/Median value	Range
Age	58 ± 9.4 yr (mean ± SD)	43–86 yr
Tumor diameter^a^	3.9 ± 1.88 cm (mean ± SD)	1.6–10 cm
LOXL4 expression score	4 (median)	1–15

AJCC, American Joint Committee on Cancer; ND, Neck Dissection; SD, Standard Deviation; LOXL4, Lysyl Oxidase-Like Protein 4.

a Tumor diameter was measured in the subset of patients treated primarily with total laryngectomy by macroscopic examination of the pathology specimen.

b 70 Gray intensity modulated radiation therapy in 35 fractions plus weekly cisplatin 35 mg/m^2^.

c Three cycles of cisplatin 75 mg/m^2^, docetaxel 75 mg/m^2^, 5-fluorouracil 750 mg/m^2^ followed by concomitant chemoradiotherapy for responding patients and salvage total laryngectomy for non-responders.

Of the 43 patients that were initially assigned to organ preservation, a salvage total laryngectomy was ultimately necessary in 17 (39%) due to induction failure or local recurrence. Mean follow-up duration was 37 months (±27.5 mo.) and 61 cases (85%) had sufficient followup data to be included in survival analysis.

**Figure 1:**
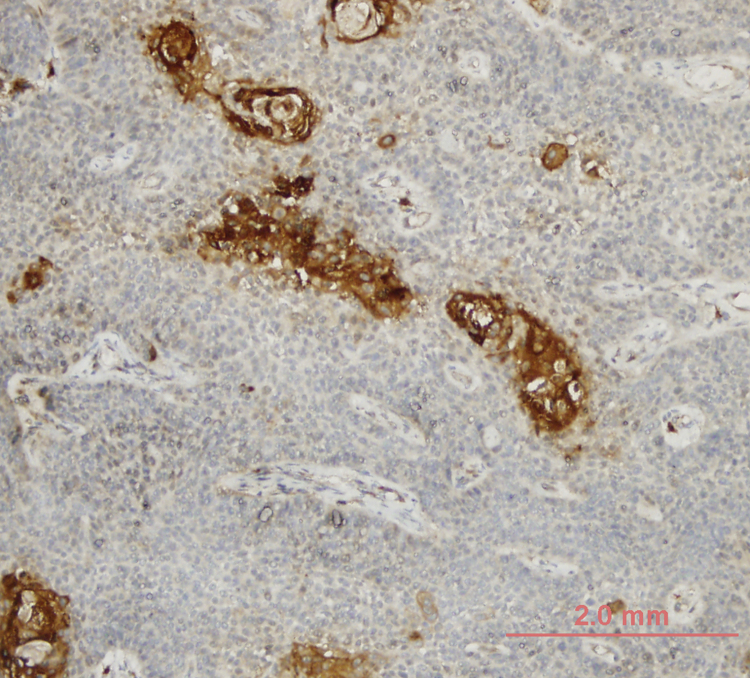
Intense LOXL4 expression in well-differentiated tumor zones.

LOXL4 expression was positive in 25 (34.7%) and negative in 47 (65.3%) tumors. Higher T stage and LOXL4 expression showed a statistically significant correlation with 15 (50%) of T4 tumors vs. 10 (23.8%) of T3 tumors considered LOXL4 positive (*p* =  0.021). A similar correlation was not evident with respect to the nodal stage of patients (*p* =  0.171). Tumors with better differentiation exhibited markedly increased LOXL4 expression as 13 (52%) of well differentiated tumors and 25 (25.5%) of moderate-poorly differentiated tumors showed LOXL4 positivity and this finding was found to be statistically significant (*p* =  0.025) (Table 2). A demonstration of intense immunohistochemical staining at well-differentiated tumor zones may be found in Fig. 1.

**Table 2:** The association of LOXL4 expression with tumor characteristics.

		LOXL4 negative	LOXL4 positive	*p-*Value
AJCC T-classification	T3	32 (76.2%)	10 (23.8%)	
	T4	15 (50%)	15 (50%)	
	Total	47 (65.3%)	25 (34.7%)	*p* = 0.021^a^
AJCC N-classification	N0	30 (68.2%)	14 (31.8%)	
	N1	6 (85.7%)	1 (14.3%)	
	N2-3	11 (52.4%)	10 (47.6%)	
	Total	47 (65.3%)	25 (34.7%)	*p* = 0.248
Stage	III	25 (78.1%)	7 (21.9%)	
	IV	22 (55%)	18 (45%)	
	Total	47 (65.3%)	25 (34.7%)	*p* = 0.041^a^
Tumor differentiation	Well	12 (48%)	13 (52%)	
	Moderate-poor	35 (57.9%)	12 (25.5%)	
	Total	47 (65.3%)	25 (34.7%)	*p* = 0.025^a^

AJCC, American Joint Committee on Cancer; LOXL4, Lysyl Oxidase-Like protein 4.

a *p*-values are statistically significant (*p* <  0.05), Pearson’s χ2 test.

**Figure 2:**
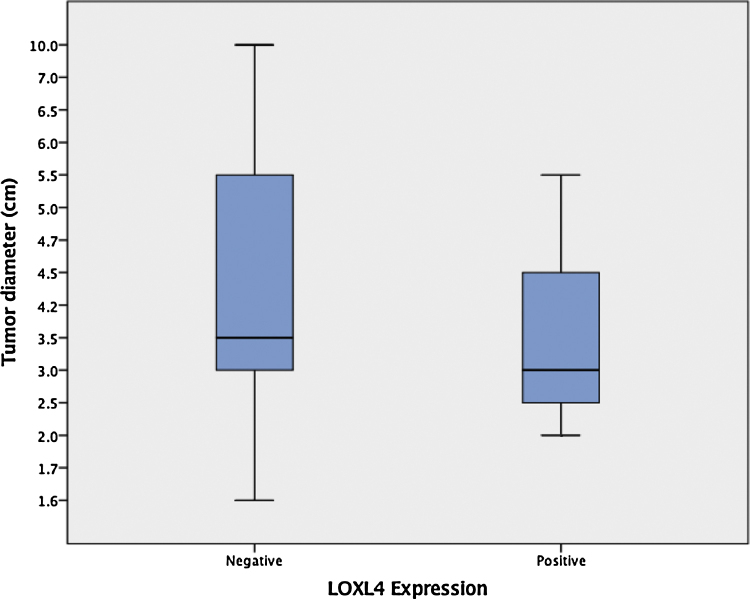
Boxplot comparison of tumour diameter with respect to LOXL4 expression. The difference between medians was not statistically significant (*p* =  0.456, Mann–Whitney *U* test).

Among 29 patients that were treated primarily with total laryngectomy, enlarging tumors had a tendency to lose LOXL4 expression (mean tumor diameter 4.1 ± 0.49 [LOXL4 negative] vs. 3.4 ± 0.38 [LOXL4 positive]) but this trend did not reach statistical significance (*p* = 0.456) (Fig. 2).

While the need for salvage laryngectomy seemed to increase in LOXL4 negative cases (76.5% had salvage laryngectomy vs. 23.5% of LOXL4 positive patients) the association was not statistically significant (*p* =  0.070). Likewise, no correlation was found between LOXL4 expression and response to induction chemotherapy (*p* = 0.999) (Table 3).

**Table 3:** The association of LOXL4 expression with induction response and organ preservation success.

		LOXL4 negative	LOXL4 positive	*p*-Value
Induction response^a^	Responsive	11 (52.4%)	10 (47.6%)	
	Unresponsive	2 (50%)	2 (50%)	
	Total	13 (52%)	12 (48%)	*p* = 0.999
Salvage laryngectomy^b^	Laryngectomy (+)	13 (76.5%)	4 (23.5%)	
	Laryngectomy (-)	10 (47.6%)	11 (52.4%)	
	Total	23 (60.5%)	15 (39.5%)	*p* = 0.070

LOXL4, Lysyl Oxidase-Like protein 4.

a Patients with ≥50% reduction in tumor diameter after three cycles of cisplatin 75 mg/m^2^, docetaxel 75 mg/m^2^, 5-fluorouracil 750 mg/m^2^ were considered responsive.

b Patients that needed salvage laryngectomy due to failed organ preservation therapy or local recurrence were included.

**Figure 3:**
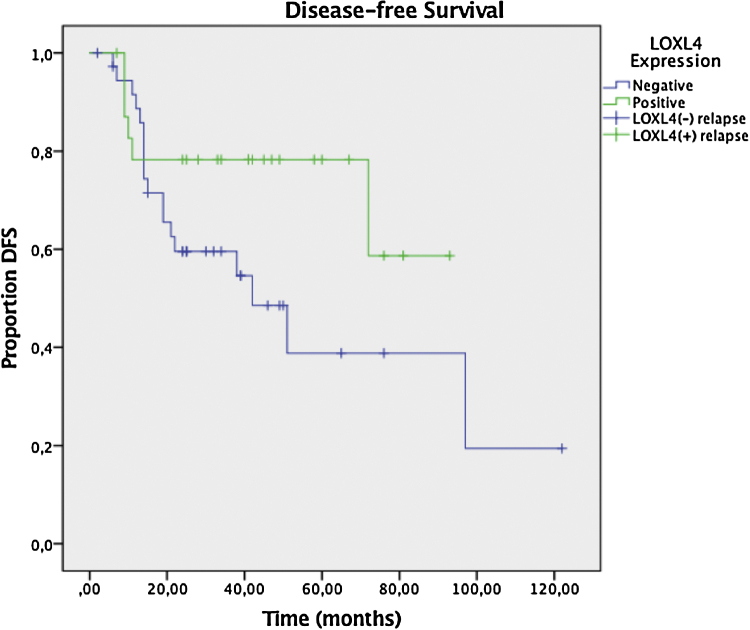
Kaplan–Meier plot for Disease-Free Survival (DFS) in LOXL4 positive and negative patients. The difference between LOXL4 positive and negative patients was not statistically significant (*p* =  0.104, Mantell-Cox log rank test).

**Figure 4:**
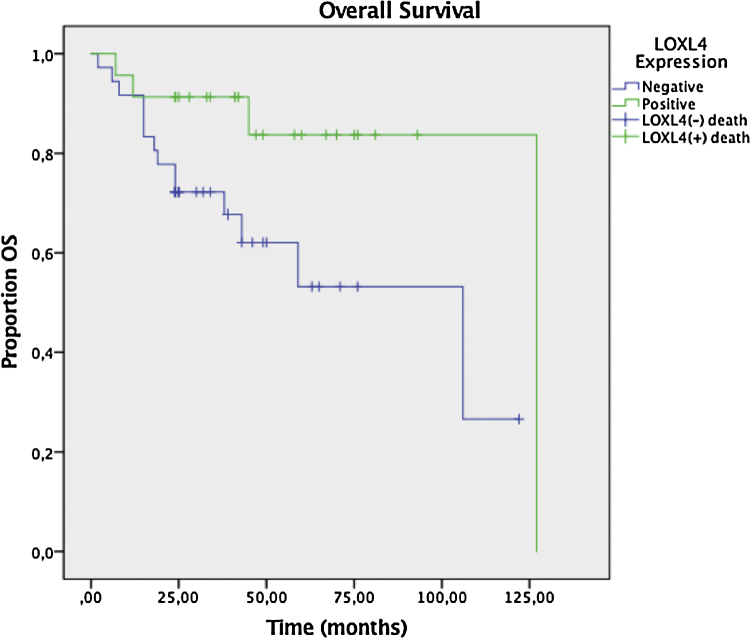
Kaplan–Meier plot for Overall Survival (OS) in LOXL4 positive and negative patients. The difference between LOXL4 positive and negative patients was statistically significant (*p* =  0.036, Mantell-Cox log rank test).

Two year DFS and OS analyses conducted by the Kaplan–Meier method in a subset of 6 patients that had sufficient follow-up data revealed a trend toward better 2 year DFS (78.3% vs. 59.6%) for LOXL4 positive patients over LOXL4 negatives, yet this finding was not statistically significant (*p* = 0.104). OS demonstrated a clearer trend toward improved survival for LOXL4 positive patients (91.3% vs. 72.2%) which was statistically significant (*p* = 0.036). The influence of various parameters on 2-year DFS and OS may be observed on Table 4. Figure 3, Figure 4 display survival curves for 2-year DFS and OS, respectively.

**Table 4:** The effect of variables on 2-year Disease-Free Survival (DFS) and Overall Survival (OS) estimated by Kaplan–Meier analysis.

			2-yr DFS			2-yr OS		
		**#**	%	95% CI	*p*	%	95% CI	*p*
LOXL4 expression	+	24	78.3	61–95	0.104	91.3	69–98	0.036^a^
	–	37	59.6	43–76		72.2	55–84	
AJCC T-classification	T3	24	72.2	53–91	0.396	81.1	64–90	0.184
	T4	37	63.9	48–80		70.8	48–85	
AJCC N-classification	N0	38	71.1	51–91	0.413	78.3	59–87	0.974
	N+	23	64.9	50–80		76.3	55–90	
Stage	III	27	74.8	60–90	0.113	79.4	62–90	0.870
	IV	34	57.7	39–77		74.1	53–87	
Tumor differentiation	Well	21	73.7	54–93	0.393	80.9	57–92	0.925
	Mod-poor	40	64	49–79		75	59–86	

DFS, Disease-Free Survival; OS, Overall Survival; CI, Confidence Interval; LOXL4, Lysyl Oxidase-Like Protein 4; AJCC, American Joint Committee on Cancer.

a *p*-values are statistically significant (*p* < 0.05), Mantell-Cox log rank test.

Next, a multivariate analysis was undertaken to ascertain that LOXL4 positivity was an independent factor for improved OS, particularly to avoid confounding with the effect of better tumor differentiation in LOXL4 positive patients. LOXL4 negativity had a hazard ratio of 5.38 (95% CI 1.4–20.7, *p* = 0.014) for death in Cox regression analysis, independent of tumor stage and differentiation.

## Discussion

Advanced stage laryngeal SCC is an aggressive malignancy of heterogeneous genetic makeup and clinical behavior that has declining 5 year survival rates. Organ preservation approaches combine radiotherapy with weekly chemotherapy to provide treatment without the need for radical surgery. Identification of patients that would benefit from this multimodality treatment, alternative adjuvant therapeutic methods and prognostication with subtyping of the tumor are recent topics of debate.

Tissue hypoxia is significant in solid tumors with a volume over 1 cm^3^ and possibly drives an aggressive/ metastatic phenotype.^9^ Hypoxia leads to a specific genomic response in human cells: A transcription factor, hypoxia inducible factor-1 (HIF-1), binds and activates hypoxia responsive elements (HREs) in promoters of certain genes that constitute the hypoxic response.^10^ Included in these upregulated proteins are quinone-containing copper amine oxidases “lysyl oxidase (LOX)” family which catalyze the oxidative deamination of lysyl residues in collagen and elastin to stabilize the extracellular matrix. Pioneering research by Erler, et al.^6^ showed that LOX expression is regulated by HIF, patients with high LOX-expressing tumors have poor survival and inhibition of LOX eliminates metastasis in mice with orthotopically grown breast cancer tumors. Yet the authors pointed out that LOX expression is paradoxically associated with both tumor progression and tumor suppression depending on cell type and transformation status, based on previous research on breast and gastric cancer cells.^11^, ^12^ In 306 patients of the RTOG 90-03 cohort (a phase 3 trial comparing different radiation fractionation series in locally advanced head and neck cancer), increased LOX expression was associated with shorter time to metastasis, time to progression and worse overall survival.^7^

LOX family has four other enzymes, LOX-like proteins 1–4 (LOXL1–4) and the last member of this family LOXL4 has been investigated for its expression characteristics and potential effects in HNSCC. LOXL4 was first proposed as a selective molecular marker for HNSCC by Gorogh, et al.^13^ in a 2007 paper that demonstrated overexpression of LOXL4 mRNA transcript in 74% of invasive HNSCC tumors and 90% of both primary and metastatic HNSCC cell lines. Scola, et al.^8^ further elucidated that compared to EGFR, an established molecular marker in HNSCC, LOXL4 overexpression may be exceedingly selective (71% in tumor tissue vs. 9% in normal mucosa). The findings in laryngeal SCC by Yilmaz, et al.^14^ are consistent with previous research in that LOXL4 was found to be strongly overexpressed in LSCC compared to corresponding normal mucosa, but this paper was not able to demonstrate a relationship between LOXL4 and tumor stage or metastatic tendency. Our findings indicate while advancing T class and stage is correlated with increased LOXL4 expression, nodal stage does not exhibit a similar association. This may suggest that LOXL4 expression is a significant factor for primary tumor progression yet not equally important for regional metastatic tendency, contrary to the findings for its parent enzyme LOX.

The association between tumor differentiation and LOXL4 expression has not been debated thus far in the English literature. A particular finding in our research is that LOXL4 expression is significantly correlated with better differentiation in LSCC. With dedifferentiation, LSCC cells may be theorized to lose the ability to express ECM modifying enzymes such as LOXL4. Increasing tumor size was expected to be another predictor for LOXL4 expression, as an enlarging solid tumor invariably becomes more hypoxic. Mean tumor diameter was found to be higher in LOXL4 positive patients, but this trend did not reach statistical significance. This fact can be explained by the smaller sample used in the analysis (patients that underwent total laryngectomy as primary treatment) due to the archival unavailability of cross-sectional imaging for all patients.

Hypoxia markers were extensively studied as prognostic indicators in solid tumors and the genomic response to tissue hypoxia termed “hypoxia signature” has been associated with poor prognosis in several types of malignancy such as breast and ovarian cancer.^15^ Laryngeal SCC is no exception, and a study by Eustace, et al.^16^ determined that tumors classified as more hypoxic according to the median expression of a 26-gene hypoxia array both had worse 5 year regional control compared to less hypoxic tumors and benefited from hypoxia modifiers carbogen and nicotinamide during accelerated radiotherapy (5 year regional control was 100% compared to 81% for radiotherapy alone). There is further research on specific hypoxia markers in advanced stage laryngeal SCC: Bernstein, et al.^5^ investigated the effect of hypoxia inducible factor-1α (HIF-1α) and CA-9 expression in 114 patients with T3-4 SCC of the larynx or hypopharynx and found worsened disease-specific survival for CA-9 positive patients. Contrary to the general trend in this subject, the single paper with prognostic implications for LOXL4 expression in HNSCC by Weise, et al.^17^ has found a tendency toward improved survival in patients that express a high level of LOXL4 in their nodal metastatic tissue, though this correlation was not statistically significant. Our prognostic results are parallel to this finding: LOXL4 positivity was associated with improved 2 year disease specific (78.3% vs. 59.6%) and overall (91.3% vs. 72.2%) survival independent of tumor differentiation, but this trend only reached statistical significance for 2 year overall survival. Research on human bladder cancer may shed light into this seemingly paradoxical situation: Wu et al.^18^ proposed that overexpression of LOXL1 and LOXL4 exhibits tumor suppressive action through inhibition of the Ras/ERK signaling pathway in bladder cancer cells. The role of LOXL4 in tumor progression in laryngeal SCC has yet to be determined clearly but our findings in light of the literature suggest that LOXL4 is an enzyme overexpressed by an advancing SCC that is lost with worsening differentiation and confers a survival advantage by hitherto undefined tumor suppressive properties.

Regardless of the conflicting findings regarding LOX family on prognosis, LOXL4 is also an important therapeutic candidate due to its highly specific expression by laryngeal SCC cells. Weise, et al.^19^ used LOXL4-transfected dendritic cells to induce a LOXL4 specific cytotoxic T-cell response in a feasibility study for cell-meditated immunotherapy. A recent paper by Gorogh, et al.^20^ remarkably demonstrated that anti-LOXL4 monoclonal antibodies significantly inhibit growth in 80% of 15 HNSCC cell lines and cause extensive tumor destruction in 41 animals xenografted with HNSCC. These results suggest that anti-LOXL4 immunotherapy could be applicable to HNSCC exhibiting tumor specific upregulation of LOXL4.

While our cohort is substantial in that it is comprised of a relatively homogeneous population of advanced stage laryngeal SCC patients that are distributed almost evenly across primary surgery and organ preservation, a retrospective design and certain shortcomings of the followup data have limited the generalizability of our conclusions. We believe that a larger, prospective cohort with fully quantitative methods to assess LOXL4 expression may yield stronger findings. The potential role of LOXL4 as a tumor suppressor at the cellular level also warrants further research to clarify the sophisticated function this novel marker has in tumor progression.

## Conclusion

Laryngeal SCC has an intrinsic heterogeneity that influences treatment response and prognosis. Subtyping with appropriate biomarkers is necessary for tailoring treatment to specific properties of the tumor, e.g., tumor hypoxia. LOXL4 is a hypoxia-activated enzyme that is highly specific for head and neck SCCs and is more strongly expressed by tumor cells in higher stages. Our research has shown that LOXL4 may also have certain tumor suppressive properties, as patients with high LOXL4 expressing tumors have longer overall survival. The exact role LOXL4 plays in SCC tumorigenesis needs further clarification, and novel treatment methods such as LOXL4 targeting immunotherapy may be grounded on these findings.

## Conflicts of interest

The authors declare no conflicts of interest.
